# On Lactation

**Published:** 1851-03

**Authors:** 


### OBSTETRICS.

*On Lactation.* By M. Trousseau.—Exhausted as this subject might seem to have become, M. Trousseau nevertheless makes several interesting observations upon it. An infinitely larger number of infants are brought up by wet-nursing in France, especially in the large towns, than with us; for not only are the mothers more occupied in business or amusement, but their medical advisers are accustomed to place more restrictions upon their acting as nurses to their own children, than we think desirable. The mother should be allowed to follow her natural inclination to nurse, M. Trousseau says, when she is strong, has a good constitution, is unaffected with hereditary di-ease, and her breasts are of a good configuration, provided she is not so placed that she is able to take little or no exercise, or cannot resign the dissipated habits of gay society.

In deciding beforehand whether a woman is likely to prove a good nurse, the *large size of the breasts* has been erroneously considered a sign of the affirmative. The mammary gland, separated from the adipose matter surrounding it, is always very much of the same size, and does not secrete milk in proportion to the size of the breast. A far better sign is drawn from the veins of the organ being very distinct and its vascularity well marked. Another, is the fact of the woman having been accustomed to perceive a vigorous flux towards the mammæ just before each menstrual epoch, the breasts then becoming hardened and the lobules of the gland as it were more prominent. When this physiological sign is distinctly present, it is the most important and most certain of all, although not indicated by preceding writers.

When a young woman becomes pregnant, her *nipples* swell and increase in length to about 4 lines, the surrounding papillæ become prominent, and the areola dark. Such a person will have a good nipple for suckling; but in a great number of women the nipple is only on a level with the breast, and, in such, means should be taken to bring it out during the latter months. The best means of effecting this is to wear a nipple-shield under the corset. If this is worn for the two or three last months, the nipple is found to have acquired the necessary projection by reason of the compression that has been exerted at its base alone.

As to the period when the child ought to be applied to the breast, M. Trousseau directs this to be a few hours after delivery, when it will very shortly procure milk, although what is called "the draught" may not take place for two or three days. If suckling be delayed till then, the breast becomes painful, the nipple buried and ill-erected, the lacteal vessels obstructed with colostrum, and suction is both difficult and painful; while engorgement and abscess of the breasts, or fissures of the nipple, may compel an abandonment of suckling altogether. [There can be no doubt of the importance of early suckling here urged by M. Trousseau, especially with the first child; but in women who suffer severely from after-pains, these are dreadfully increased during suckling, so as often to necessitate its postponement.] With respect to the child, M. Trousseau advises an ingenious procedure which we are inclined to think might have saved trouble in some cases we have seen. It is the giving it to suck, before applying it to the breast, a very short hard piece of linen, tied up in the shape of a nipple, and moistened with a little sugar and water. The infant likes the taste, but the body being hard and short, it finds, after its efforts upon it, that the suction of the mother's nipple is a much more pleasant operation. It has already tried its strength against a greater resistance, and without having done so it will often not take the nipple kindly, The fluid employed must not be too sweet, or the mother's milk will appear tasteless afterwards.

As to *regulation of the suckling*, this must not be attempted at first. Children in hospitals lose weight during the first nine days, andin private life during the first two days, and at first they should be allowed to suck as much as they will, which is also of use to the mother, who has then abundance. This may go on during the fortnight or month that she is within doors, and can sleep or rest as she likes; but when she resumes her occupations, suckling should be regulated, and above all things so as to allow rest at night. To this end, during the first two or three months, the child should be suckled at short intervals during the day, but only once between 8 and 9 at night and 7 next morning. After three or four months, most women require a little assistance; and a meal of milk mixed with any suitable fluid should be given the child by the sucking bottle at the beginning or middle of the night, by which means the mother's repose and the reaccumulation of her milk are aided. During the day artificial food must at first be chiefly formed of milk or farinaceous matters, by far the best of these being, in spite of Rousseau's denunciations, *pap*, made with the crumbs of well-baked, light stale bread. It is of importance that the food should not always be the same but composed of different farinaceous articles, &c., at different meals, and care being taken to observe which is digested best. As the time for weaning approaches, the food must be more varied, eggs differently prepared, chicken and other broths, and especially beef-tea should be employed, still however, encouraging the infant's taste for milk.

In respect to the *nurse*, of course she should observe all the rules dictated by a sound hygiene, if she wishes to preserve her milk good. The most absurd prejudices prevail in respect to nourishment; and thus it often happens, that a nurse arriving from the country, who had enjoyed excellent health on a vegetable diet, becomes ill and loses her milk through the highly animalized food she is overloaded with. If a nurse menstruates, 99 out of 100 mothers would change her, although the milk is as good, but in a smaller quantity just at the epoch. Most women after 11 or 12 months menstruate without their milk deteriorating. The case may be different if menorrhagia or leucorrhœa is present. M. Trousseau also considers that the milk, though lessened in quantity, is not deteriorated by the occurrence of a new pregnancy. About the sixth month, however, becoming mingled with colostrum, it purges the child a little, and then weaning should be resorted to. He has very frequently seen a nurse able to suckle quite well until her confinement, although he certainly would not choose one if he knew she was pregnant.

In towns *bringing up children by hand* is a most difficult matter, and never to be advised. In the provinces, where abundance of good milk can be got, it is far easier, and in Normandy almost all the children are so brought up. Even there, however, a great number perish. Among all the varieties of sucking bottles, the best is a phial, corked by a piece of sponge, and tied over by a piece of old linen, the fluid always being given at the temperature of the body. It is necessary that the child should make some effort in sucking, or saliva is not educed. Instead of the linen, a piece of chamois leather answers well, if kept constantly well washed.

*Weaning* should be delayed as long as possible, provided neither nurse or child suffer, and in the case of weakly children even for two or three years. This is the epoch of their dentition and various maladies, and it is very convenient to have so digestible an aliment at one's command. It is to be observed, too, that the longer they continue to suck, the less do they entirely depend on the breast. Age should not guide us in determining upon weaning, so much as the evolution of the teeth. This evolution takes place in *groups*. In the first group, the two lower median incisors appear, in the second the four upper incisors, in the third the four first molars and two lower lateral incisors, in the fourth the four canines, and in the fifth the four last molars. During the evolution of these respective groups, weaning should never be attempted; while, after this is completed, an interval of repose favorable to the attempt occurs. It is not until the canines have appeared that we should advise weaning, if avoidable, these being the teeth attended with the most difficulty in their evolution. The child before weaning should have been gradually accustomed to other articles of diet; and in those cases in which it seems to have an aversion to milk, every variety of food must be tried till the suitable one is found. There is, in fact, no rigorous rule upon this subject; but that should be given which is best digested.—*Brit. and For. Rev. from Gazette des Hôpitaux*, Nos. 23 and 24.

